# Accounting for non-response bias using participation incentives and survey design: An application using gift vouchers

**DOI:** 10.1016/j.econlet.2018.07.040

**Published:** 2018-10

**Authors:** Mark E. McGovern, David Canning, Till Bärnighausen

**Affiliations:** a CHaRMS — Centre for Health Research at the Management School, Queen’s University Belfast, Belfast, Northern Ireland, United Kingdom; b Centre of Excellence for Public Health (Northern Ireland), United Kingdom; c Africa Health Research Institute, Somkhele, South Africa; d Harvard T. H. Chan School of Public Health, Boston, USA; e Institute of Public Health, Faculty of Medicine, Heidelberg University, Heidelberg, Germany

**Keywords:** Participation incentives, Survey design, Selection bias, Non-ignorable missing data, Selection models, HIV

## Abstract

Standard corrections for missing data rely on the strong and generally untestable assumption of missing at random. Heckman-type selection models relax this assumption, but have been criticized because they typically require a selection variable which predicts non-response but not the outcome of interest, and can impose bivariate normality. In this paper we illustrate an application using a copula methodology which does not rely on bivariate normality. We implement this approach in data on HIV testing at a demographic surveillance site in rural South Africa which are affected by non-response. Randomized incentives are the ideal selection variable, particularly when implemented ex ante to deal with potential missing data. However, elements of survey design may also provide a credible method of correcting for non-response bias ex post. For example, although not explicitly randomized, allocation of food gift vouchers during our survey was plausibly exogenous and substantially raised participation, as did effective survey interviewers. Based on models with receipt of a voucher and interviewer identity as selection variables, our results imply that 37% of women in the population under study are HIV positive, compared to imputation-based estimates of 28%. For men, confidence intervals are too wide to reject the absence of non-response bias. Consistent results obtained when comparing different selection variables and error structures strengthen these conclusions. Our application illustrates the feasibility of the selection model approach when combined with survey metadata.

## Highlights

• Standard missing data approaches like imputation assume that data are missing at random (MAR).
• There are many contexts in which this MAR assumption is implausible.
• Heckman-type selection models can be used to test for MAR.
• Robustness to alternative selection variables and dependence structures strengthens the credibility of results.
• Randomized incentives or survey interventions provide ideal selection variables.

## 1 Introduction

Because of the implications for estimation, adjusting for missing data is an important component of program evaluation. Missingness can arise in various contexts including attrition in panel surveys (Thomas et al., 2001), mortality (Attanasio and Hoynes, 2000), and declining to answer particular survey questions or participate in auxiliary health or biomarker modules (Lillard et al., 1986). Adjustments are especially problematic because by definition we do not observe outcomes for non-respondents, so missing data mechanisms are generally not directly testable (Nicoletti, 2006). This has contributed to a reliance on methods which assume missing at random (MAR), often conditional on observables. These approaches include imputation (Conniffe and O’Neill, 2011), and inverse-probability weighting (Wooldridge, 2007). However, there are many contexts in which MAR may not be realistic.

Alternative selection model approaches (Heckman, 1979), simultaneously specify participation alongside the outcome without requiring MAR. However, Heckman-type selection models have been criticized because alternative assumptions are necessary (Vytlacil, 2002). First, in practice an exclusion restriction is required, a variable which predicts participation but not the outcome. Plausible selection variables can be elusive (Madden, 2008), but model performance depends on their validity (Leung and Yu, 1996). Second, they can require parametric assumptions. The original formulation specified the joint distribution of the error terms in participation and outcome equations as bivariate normal. Extensions incorporate binary outcomes (Van de Ven and Van Praag, 1981) and semiparametric and nonparametric variants (Ahn and Powell (1993), Das et al. (2003), Gallant and Nychka (1987), Newey et al. (1990)). The latter have larger data requirements and are less efficient than their parametric counterparts. Moreover, the intercept is often the quantity of interest (Heckman, 1990), and estimating the intercept in semiparametric or nonparametric selection models generally focuses on continuous outcomes and requires additional identification at infinity assumptions (Andrews and Schafgans (1998), Schafgans and Zinde-Walsh (2002)). Reduced information inherent in binary (relative to continuous) data precludes estimation of the intercept without parametric restrictions (Klein et al., 2015).

Therefore, there is a trade-off between two sets of assumptions when attempting corrections for non-response. Lacking viable selection variables, it is understandable that researchers would proceed on the basis of MAR, even if objectively implausible. Alternative bounding approaches can be useful for avoiding this trade-off (Behaghel et al. (2015), Lee (2009), Manski (1990)), but may not be informative when rates of non-response are high, resulting in too wide a range of possible estimates. Improving the methodology for implementing selection models therefore provides opportunities to avoid having to assume MAR.

Although well known that (quasi) experimental manipulation can solve for endogenous sorting into treatment groups, (quasi) experiments can also be used for dealing with non-response. Survey design affects participation (Hirano et al. (2001), Hill and Willis (2001)), and these findings have informed methods to reduce measurement error (Gibson et al., 2015). The resulting impact on participation has also been used to adjust for non-response bias, as these features of survey design can be used as selection variables in a Heckman-type framework. For example, Bhattacharya and Isen 2008 use a $5 gift certificate randomized to a subset of student respondents to adjust for non-response in a survey on willingness to pay for health care. Bailey 2017 examines sample selection in political surveys by randomly allocating some participants to a condition in which the political questions are asked after those on another topic. Interviewer identity is another selection variable which has been used to adjust for panel attrition (Van den Berg et al., 2007) and missing data in biomarker data (Reniers et al., 2009; Tchetgen and Wirth, 2017).

The ideal selection variable in this context is a randomized incentive or survey intervention because it is guaranteed to be unrelated to the outcome (in expectation) other than through any effect on participation. Because this approach is relatively rare, there are not many opportunities to leverage randomization to correct for missing data. However, there may be elements of survey design which are as good as random in some survey contexts ad therefore provide credible selection variables enabling this approach to be adopted more widely.

The contribution of this paper is to apply this methodology to data on HIV testing from demographic surveillance in South Africa, comparing standard approaches which assume MAR to selection model estimates. We adopt the copula-based framework developed in Marra et al. 2017 which allows flexible specification of unobserved dependence using various distributional forms. We build on this analysis by illustrating an application using two selection variables based on survey design; a food gift voucher and interviewer identity, which although not randomized, are plausibly exogenous in this survey context. We argue that showing results are robust to alternative exclusion restrictions and different distributional assumptions, as this framework allows, strengthens the conclusions from selection models.

## 2 Non-response in HIV research

Non-response is particularly concerning when there is an incentive not to participate. For example, people who are HIV positive may systematically opt out of testing because they fear disclosure of their status (Obare, 2010). However, accurate estimates of HIV prevalence are important because they provide information about the spread of the epidemic (Beyrer et al., 1999) and facilitate intervention evaluation (Baird et al., 2010).

Nationally representative household surveys and surveillance sites routinely include blood tests, and resulting prevalence estimates are considered the gold standard (Boerma et al., 2003). However, in some contexts less than half of eligible respondents participate (Larmarange et al., 2015). Trials are also affected; of 57 RCTs conducted before 2012 with HIV status outcomes, missing data ranged from 3% to 97% (mean 26%), with no study reporting their assumptions for managing non-response (Harel et al., 2012). Given the potential for HIV positive individuals to be systematically less likely to participate (Arpino et al., 2014), imposing an incorrect MAR assumption could result in substantial bias.

## 3 Participation incentives and survey design as selection variables

The Africa Health Research Institute (AHRI) cohort is a continuous survey of residents of a rural area in KwaZulu-Natal, South Africa. The main survey and HIV surveillance have provided valuable information on the epidemic for over a decade. Table 1 demonstrates that 45% of women participated in testing in 2010; compared to 33% of men. HIV prevalence among these participants was found to be 27% (women) and 16% (men). Potential implications of non-response are clear from nonparametric bounds, which, in this case, are too wide to be informative. In this paper we use the terms participation and consent to test interchangeably as relatively few individuals decline to participate in the survey (before consent to test for HIV is sought), but in other contexts they may need to be considered separately. Further information about the survey and cohort are presented in the supplementary material.

To increase participation, a gift voucher intervention was conducted in 2010. During the last 10 weeks of the surveillance, interviewers presented potential respondents with a food voucher worth 50 South African Rand to the first person they met in each household. 7% of those contacted in 2010 received a voucher, which was not conditional on consent. While not randomized, the intervention reflected concern among management about low participation in the first half of the surveillance, and apart from the timing, was not otherwise targeted. Previous evaluation found the gift voucher successfully raised participation by 25 percentage points (PP) (McGovern et al., 2016). Almost all those who received the voucher were living in households that were contacted in October or November. Once month of interview is controlled for, there is little evidence that the characteristics of those who received the voucher differ from those who did not receive the voucher (as shown Table A3 in the supplementary material). A joint test of covariates other than month yields an F statistic of 0.80, p=0.88. Although this does not conclusively rule out a role for unobserved factors, and results should be interpreted with this in mind, it does provide some support for the hypothesis that the gift voucher was as good as randomly distributed (conditional on the timing of the interview). Given this, we use gift voucher receipt as a selection variable alongside an alternative based on survey design, interviewer identity. The interviewer a person has been allocated often strongly affects participation (Thomas et al., 2012), including in HIV testing (Janssens et al., 2014).

The assumption that interviewers are as good as randomly allocated may not always be appropriate. However, at AHRI, interviewers operate in teams who move from district to district as dictated by managers, attempting to contact all eligible households, and therefore the interviewer a respondent is allocated depends on survey procedure and is unlikely to be correlated with unobserved characteristics. Table A2 in the supplementary material presents results of a regression of interviewer success (which we define here as the proportion of each interviewer’s interviewees who consent to test) on interviewee characteristics. However, because interviewer success will depend not only on interviewee characteristics but also on unobserved characteristics of the interviewer, it is difficult to interpret these associations. Nevertheless, we can assess the plausibility of the exclusion restriction indirectly by comparing results from two different selection variables.

**Fig. 1:**
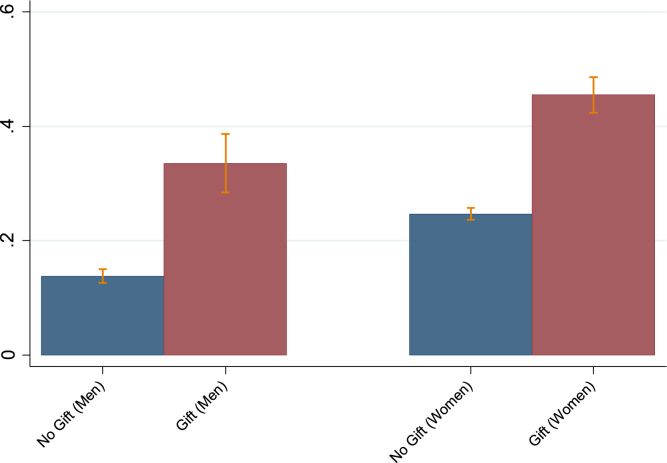
AHRI 2010 HIV Prevalence by Gift Voucher Receipt. Note: For each group (received a gift voucher or did not receive a gift voucher), the HIV prevalence rate is calculated as the number of HIV positive respondents among those who participated in testing in that group divided by the number of respondents who participated in testing in that group. 95% confidence intervals are shown.

Fig. 1 shows mean HIV prevalence (among participants) for AHRI residents in 2010 according to whether they received a voucher. Assuming gift receipt was exogenous, higher prevalence among those who received the incentive suggests those who would ordinarily refuse to test, but were persuaded by the gift voucher to test in this case, are more likely to be HIV positive. This is preliminary evidence of non-response bias as it indicates those who tend to decline to participate are more likely to be HIV positive.

**Table 1:** Participation in HIV Testing and HIV Prevalence at the 2010 AHRI Surveillance Cohort.

	Women			Men	
	No.	%		No.	%
Refused to Test	9,357	55		7,210	67
					
Consented to Test	7,590	45		3,527	33
					
Total	16,947	100		10,737	100

	Women			Men	
		%			%

Did not receive gift voucher — consented to test		42			31
					
Received gift voucher — consented to test		58			41

	Women			Men	

HIV Prevalence (%)	27			16	
					
95% CI for Nonparametric Bounds	12	68		5	73

					

Note: HIV prevalence estimates are based on those who participated in testing. Confidence intervals for nonparametric bounds are based on Horowitz and Manski 2006.

## 4 Model

The standard selection model (Heckman, 1979) specifies participation and the outcome simultaneously as a function of covariates and a selection variable which enters only into the participation equation. For binary outcomes the model is a bivariate probit based on latent variables (Van de Ven and Van Praag, 1981):

(1) $$Consent_{i}^{*}=X_{i}^{T}\beta_{c}+Z_{j}^{T}\gamma +u_{i},i=1,\ldots ,n,\;j=1,\ldots ,J$$

(2) $$Consent_{i}=1\text{ if }Consent_{i}^{*}>0,Consent_{i}=0\text{ otherwise},$$

The latent variable for whether person i with interviewer j consents to test, $Consent_{i}^{*}$, depends on individual and household characteristics, $X_{i}$, and interviewer effects, $Z_{j}$. When using the gift voucher, a vector indicating receipt replaces the interviewer effects. In addition, there is a random error term, $u_{i}$.

Similarly, the latent variable for the HIV status of person i with interviewer j, $HIV_{i}^{*}$, is given by:

(3) $$HIV_{i}^{*}=X_{i}^{T}\beta_{h}+\epsilon_{i}$$

(4) $$HIV_{i}=1\text{ if }HIV_{i}^{*}>0,HIV_{i}=0\text{ otherwise},$$

Where $X_{i}$ is the same matrix of covariates included for $Consent_{i}^{*}$, and $\epsilon_{i}$ is a random error term. The selection variable, $Z_{j}$, does not enter into the outcome equation. We only observe HIV status conditional on consent:

(5) $$HIV_{i}\text{ observed only if }Consent_{i}=1,\text{ missing otherwise}.$$

Previous implementations have assumed the joint error distribution is bivariate normal, $F\left(\epsilon_{i},\mu_{i}\right)=\Phi_{2}\left(\epsilon_{i},\mu_{i};\rho \right)$, where $\Phi_{2}$ is the standardized bivariate normal cumulative density function (CDF): $\Phi_{2}\left(\epsilon_{i},\mu_{i};\rho \right)=\int_{-\infty }^{\mu }\int_{-\infty }^{\epsilon }\frac{1}{2\pi \sqrt{1-\rho^{2}}}e^{\frac{1}{2\sqrt{1-\rho^{2}}}\left(s^{2}+t^{2}-2st\rho \right)}dsdt$. If the true error structure does not meet this assumption the model is misspecified, and estimates will be inconsistent (De Luca, 2008). In this paper we follow the methodology developed by Marra et al. 2017, who apply selection models to estimating HIV prevalence in three countries in sub-Saharan Africa. As described further in the supplementary material, we model the joint distribution of $\epsilon_{i}$ and $u_{i}$ using copulae, a tractable way of specifying unobserved dependence structures while allowing margins to take a variety of different forms. Using this approach, Marra et al. 2017 find evidence of non-response bias in Zambia and Swaziland.

Dependence can be incorporated into the likelihood function through relevant copula functions, for example, $p_{11i}=P\left(Consent_{i}=1,HIV_{i}=1\right)=C\left(\Phi \left(X_{i}^{T}\beta_{c}+Z_{j}^{T}\delta \right),\Phi \left(X_{i}^{T}\beta_{h}\right);\theta \right)$, where Φ is the probability density function (PDF) of the bivariate normal distribution, defined as$\frac{1}{2\pi \sigma_{1}\sigma_{2}\sqrt{1-\rho^{2}}}e^{\frac{-1}{2\left(1-\rho^{2}\right)}\left[{\left(\frac{x-\mu_{1}}{\sigma_{1}}\right)}^{2}-2\rho \left(\frac{x-\mu_{1}}{\sigma_{1}}\right)\left(\frac{y-\mu_{2}}{\sigma_{2}}\right)+{\left(\frac{y-\mu_{2}}{\sigma_{2}}\right)}^{2}\right]}$. The bivariate probit is equivalent to the Gaussian copula, with C then given by $C_{g}=\Phi_{2}\left(\Phi^{-1}\left(HIV\right),\Phi^{-1}\left(Consent\right);\theta \right)$, where $\Phi^{-1}$ is the quantile function of the standard univariate normal distribution.

Other copula applications have included selection models with continuous outcomes (Smith, 2003), and recursive models (Dancer et al. (2008), Murteira and Lourenço (2011), Prieger (2002), Winkelmann (2012)). We show results from the standard (bivariate normal) selection model along with those based on the copula selection model. An advantage of the copula approach is that it can be easily incorporated into the maximum likelihood framework, which allows the application of standard measures of model fit. As the Gaussian copula (which represents the bivariate normal case) is symmetric, this initial model can be used to identify the direction of dependence (using for instance the gamma rank association measure). If the association between error terms is estimated to be negative, as in this paper, this suggests a number of candidate copula (such as the Clayton or Joe rotated 90 or 270 degrees, as well as the Frank, which is symmetric). To determine which of these dependence structures best describe the data at hand, we identify the copula with the best fit as measured by the Akaike Information Criterion (AIC), with the lowest indicating the preferred model. In principle, other measures of fit, such as the Bayesian information criterion (BIC) could also be used for this purpose.

**Table 2:** Results for HIV Prevalence (Women).

Model	Selection Variable	HIV Prevalence	95% CI		Gamma Association	Copula
Complete Case		27	26–28			
Imputation		28	28–29			
						
Normal Selection Model	Interviewers	35	31–39		−0.42	Gaussian
Normal Selection Model	Gift voucher	33	23–42		−0.26	Gaussian
Normal Selection Model	Interviewers + Gift voucher	35	31–39		−0.40	Gaussian
						
Copula Selection Model	Interviewers	37	33–41		−0.49	Frank
Copula Selection Model	Gift voucher	39	31–47		−0.54	Frank
Copula Selection Model	Interviewers + Gift voucher	37	33–41		−0.46	Frank

						

Note: The following variables are included as predictors of consent to test for HIV and HIV status: age, month of interview, location of residence, urban/rural/peri-urban type of residence, distance to nearest clinic, distance to nearest secondary school, distance to nearest primary school, distance to nearest level 1 road, distance to nearest level 2 road, marital status, education, mother/father alive, electricity in home, fuel in home, toilet in home, water in home, and household asset index. The first row is the mean prevalence among the sample who consent to test and have a valid HIV test (complete case analysis). The second row imputes HIV prevalence for those who refused to test using the covariates described above. Row 3 implements a Heckman selection model for HIV status and consent to an HIV test using interviewer fixed effects. In row 4 the Heckman selection model uses a binary indicator for whether the respondent received the food gift voucher. The model in row 5 uses interviewers and the gift voucher intervention as exclusion restriction variables. 94 respondents who consented to test for HIV, but received indeterminate results were excluded from the procedure for estimating HIV prevalence. The copula model shown is the model with the best fit, as defined by the Akaike Information Criterion (AIC). Tables show the gamma rank association measure in column 5.

**Table 3:** Results for HIV Prevalence (Men).

Model	Selection Variable	HIV Prevalence	95% CI		Gamma Association	Copula
Complete Case		16	14–17			
Imputation		17	17–18			
						
Normal Selection Model	Interviewers	20	14–26		−0.22	Gaussian
Normal Selection Model	Gift voucher	28	12–43		−0.55	Gaussian
Normal Selection Model	Interviewers + Gift voucher	21	16–27		−0.29	Gaussian
						
Copula Selection Model	Interviewers	21	16–25		−0.20	Joe 90
Copula Selection Model	Gift voucher	33	19–46		−0.68	Frank
Copula Selection Model	Interviewers + Gift voucher	21	17–25		−0.24	Joe 90

						

Note: The following variables are included as predictors of consent to test for HIV and HIV status: age, month of interview, location of residence, urban/rural/peri-urban type of residence, distance to nearest clinic, distance to nearest secondary school, distance to nearest primary school, distance to nearest level 1 road, distance to nearest level 2 road, marital status, education, mother/father alive, electricity in home, fuel in home, toilet in home, water in home, and household asset index. The first row is the mean prevalence among the sample who consent to test and have a valid HIV test (complete case analysis). The second row imputes HIV prevalence for those who refused to test using the covariates described above. Row 3 implements a Heckman selection model for HIV status and consent to an HIV test using interviewer fixed effects. In row 4 the Heckman selection model uses a binary indicator for whether the respondent received the food gift voucher. The model in row 5 uses interviewers and the gift voucher intervention as exclusion restriction variables. 94 respondents who consented to test for HIV, but received indeterminate results were excluded from the procedure for estimating HIV prevalence. The copula model shown is the model with the best fit, as defined by the Akaike Information Criterion (AIC). Tables show the gamma rank association measure in column 5.

## 5 Results

HIV prevalence estimates are presented in Tables 2 (women) and 3 (men). Point estimates and confidence intervals based on respondents who consent to test (row 1), and an imputation (chained equations) model based on observed characteristics (row 2) are shown. We compare these to the standard bivariate normal and copula selection models. In rows 3 and 6, interviewer identity is the selection variable, while for rows 4 and 7 it is the gift voucher intervention. Rows 5 and 8 show estimates based on using both. All models include covariates and are stratified by sex to allow for differential selection effects.

Selection model estimates among women are substantially higher than imputation (28%). Point estimates are comparable using each of the selection variables, both together, and in normal and copula selection models. The Frank copula is the best fit for all models for women. Our new point estimate based on the copula selection model and both selection variables (37%) is 9 percentage points higher than the imputation model, a relative increase of 32%. There is therefore evidence that HIV positive women are less likely to participate.

For men, the evidence is less clear. Imputation estimates (17%) are lower than for normal and copula selection models using both exclusion restriction variables (21%). This 4 percentage point difference corresponds to a relative increase of 24% over imputation. However, confidence intervals are wide, which is likely to indicate too much uncertainty to rule out no selection bias, although a formal test would be required to assess the degree of statistical significance. These results are supported by analysis of the 2009 survey based on interviewers which also suggested non-response bias (McGovern et al., 2015).

## 6 Conclusions

Most standard approaches for dealing with missing data rely on assuming MAR, which may not be realistic if there are reasons to suspect participation is correlated with outcomes after controlling for observed characteristics. Previous research has shown that survey design can have a strong impact on participation (Hurd and Rohwedder, 2009). Here, we build on the flexible selection methodology developed in Marra et al. 2017 by demonstrating an application of how factors such as participation incentives or interviewer identity can be used to test for non-response bias. When survey metadata are combined with a copula approach, the usual assumption of bivariate normality can be relaxed, allowing for a wide variety of parametric distributions for characterizing the unobserved relationship between participation and outcome.

Our results illustrate the importance of testing the crucial assumption of MAR when non-response is substantial. Using data on HIV status, our prevalence estimates for the imputation-based MAR approach were almost identical to ignoring the missing data. For women, selection model point estimates indicate substantial non-response bias. For men, confidence intervals are overlapping and likely to be too wide to reject the hypothesis of no selection bias. However, if precisely estimated, the selection model point estimates would indicate a proportionally similar amount of non-response bias to women. Sex differences in participation in HIV testing and non-response are consistent with the hypothesis that women are more adversely affected by HIV status disclosure in this community. Given that the selection variables (interviewer identity and the gift voucher) are equally predictive of participation for men and women (i.e. women do not respond more strongly to the selection variable), this may suggest that HIV status may be a less important predictor of participating in HIV testing for men. Alternatively, overall consent rates for men are lower, which makes it harder to adjust for missing data using selection models, potentially resulting in wider confidence intervals. More efficient estimates based on additional data would be required to provide more concrete evidence on these hypotheses.

Other extensions of the model would also be interesting to pursue. For example, while the focus here has been on estimating the model intercept, the same framework can be adopted to examine the association between a predictor and an outcome. In the selection model, the association between household wealth and HIV status is weaker than that suggested by analysis of only those who participated. For example, among women being in the fifth household asset index quintile (compared to the first) is associated with a reduction of 6 percentage points in the probability of being HIV positive in the probit model, whereas it is smaller in magnitude (4 percentage points) and not statistically significant in the selection model. Similarly, when applied to an RCT affected by attrition, this approach can be used to estimate the selection-bias adjusted causal effect of the treatment. An important addition would be the development of a formal test assessing whether point estimates from alternative selection models with different selection variables, or other models assuming MAR, are statistically different. The equivalent of a Durbin–Wu–Hausman test (Nakamura and Nakamura, 1981) would be very useful in this context.

In the application in this paper we find very little difference between standard bivariate normal and copula selection models. However, as the true error structure is never observed, it is not possible to say whether this would be the case in other contexts. Given that the reliance on bivariate normality is commonly raised as a drawback of selection models (Bhattacharya and Isen (2008), Vytlacil (2002)), the copula approach may provide valuable sensitivity analyses of alternative dependence relationships. Comparing estimates obtained using different exclusion restrictions can strengthen the credibility of results, as can demonstrating that results are not sensitive to any one parametric specification through flexible modeling of error structures.

Missing data correction is often implemented ex post, in which case the ideal randomized selection variable is unlikely to be available. Here we have used selection variables which we believe to be credible in our context, however, in the absence of randomization, careful case by case assessment of the exogeneity assumption will be necessary. Instead, if randomization of incentives and follow-up in surveys and trials could be built into the design ex ante, this would further strengthen the credibility of missing data and attrition adjustments based on the approach we outline in this paper.

## Appendix A Supplementary data

MMC S1 Appendices file for on line publication.
