# Supplementary material for: Accounting for non-response bias using participation incentives and survey design: An application using gift vouchers
Source: Econ Lett. 2018 Oct;171:239–44. doi: 10.1016/j.econlet.2018.07.040 (PMC6167756; doi:10.1016/j.econlet.2018.07.040)
Supplement: MMC S1 — Appendices file for on line publication. [file mmc1.pdf]

Accounting for Non-Response Bias using Participation Incentives and Survey Design: An Application Using Gift Vouchers

Mark E. McGovern, David Canning, Till Bärnighausen

Appendix

Selection Bias in HIV Testing

Most of what we know about the impact and spread of the HIV epidemic in low and middle income countries comes from data collected from blood tests taken from respondents in nationally representative household surveys and surveillance sites which track the residents of specific geographic areas. Household surveys in countries without developed health service infrastructure often include routine blood draws at the end of their standard interviews. For example, this type of testing is conducted in many of the Demographic and Health Surveys (Fabic et al., 2012). These representative datasets are important because they facilitate estimation of HIV prevalence and the change in HIV prevalence over time. These estimates are required for policy as they provide information about the spread of the HIV epidemic (Beyrer et al., 1999), allow for targeting of at risk communities (Tanser et al., 2013), inform about the factors which are protective against infection (De Walque, 2007; Case and Paxson, 2013), illustrate the impact of HIV and AIDS on government services and economic growth (Bloom and Mahal, 1997; Case and Paxson, 2011), and are used to evaluate the population effectiveness of HIV interventions (Baird et al., 2010). Data obtained from testing in HIV surveillance surveys have therefore been highly influential for informing our understanding of the HIV epidemic. However, they have the drawback that rates of participation in testing in these surveys can be low. Studies which use imputation to correct for this missing data tend to find little difference between imputed estimates and estimates based on analysis of cases without missing data (Mishra et al., 2008).

A key question is therefore whether the assumption of missing at random is reasonable in this context. Unfortunately, there are three reasons to be skeptical. First, respondents who are asked to provide blood for an HIV test have an incentive to decline to participate if they know or suspect they are HIV positive because the potential costs of disclosure are high (Parker and Aggleton, 2003). Second, when those who decline are asked to explain why, a high proportion give reasons related to having been tested previously or already knowing their status (Kranzer et al., 2008). Third, there are occasional opportunities to observe longitudinal information on HIV testing. These data support the hypothesis that those who are HIV positive are less likely to participate in testing (Obare, 2010; Bärnighausen et al., 2012). For example, data from Malawi found that HIV positive residents were more than 4 times more likely to refuse to test (Reniers and Eaton, 2009). Previous research based on Heckman-type selection models and cross-sectional data has also found evidence of selection bias in some HIV surveys (Reniers et al., 2009; Janssens et al., 2014).

Data

The Africa Health Research Institute (AHRI) cohort is a continuous survey of residents of a rural area approximately 434km^2 in KwaZulu-Natal, South Africa, which has been conducted since 2003. Regular HIV testing of the residents takes place, of whom there are around 90,000 in any given year. This predominantly Zulu-speaking region remains one of the poorest in South Africa, and has experienced very high rates of HIV prevalence, along with recent scale-up of antiretroviral treatment in the locality. The data have been highly influential in informing understanding of the impact of HIV and AIDS on individuals, families, and communities, and are publicly accessible (after registration) from www.ahri.org.

As part of the main survey, data are collected on a semi-annual basis from a key informant in each household (Tanser et al., 2008). The topics covered include the characteristics of individual household members and important events in their lives (such as births, deaths, and migration), the attributes of the household (such as assets and facilities), as well as data on the physical structures themselves. The HIV surveillance cohort is nested within the main household survey and is conducted on a subset of residents (since 2007 every resident aged 15 and over has been eligible). In some years of the surveillance (before 2007 and in 2009) results are made available to those who tested, however as very few residents attempt to obtain information on their HIV status through the surveillance testing, results are not provided to surveillance participants in all years, including in 2010. This community already has very good access to rapid HIV testing and results through public-sector HIV counselling and testing. When the annual HIV surveillance is in progress, those residents who are eligible are visited by teams of two trained interviewers. In accordance with WHO and UNAIDS guidelines, these interviewers approach potential participants and seek written consent for them to obtain a blood sample. If consent is given, they prepare a dried blood spot sample collected by finger prick. If eligible individuals are not present at the household when interviewers attempt to contact them for participation, the team makes three follow-up attempts to contact the individual by revisiting the household. Once a dried blood spot is obtained, there is no identifying information for that sample, only a unique numerical code is retained to link the HIV test result with the combined HIV surveillance and household surveys datasets.

Because the main survey collects information from one key informant per household, participation in the main survey is almost universal. However, the HIV surveillance survey collects information from individuals, and a limitation of the surveillance data is that participation rates are low (Larmarange et al., 2015), and participation rates are low mainly because residents decline consent for a blood test rather than not being found for contact. Given the evidence we discuss above, this raises concerns about the accuracy of estimates based on either analysis of cases without missing data (i.e. only residents who participate in testing, ignoring those who do not participate) or imputation. In this paper we focus on the 2010 HIV surveillance because during this year a participation incentive was offered to a subset of residents. In 2010, 40,789 residents were identified from the Africa Center database as being eligible for participation in the HIV surveillance. Of these, 7,400 were found to have migrated, become sick or disabled, or had died when consent was sought. A further 5,611 residents were found to be ineligible or could not be found due to incorrect demographic or contact information. Only 186 residents declined to participate in the surveillance before being asked to take a HIV test. 27,684 individuals were successfully contacted to participate in HIV testing and had a valid test result (a small number had an indeterminate reading). Descriptive statistics are shown in Table A1.

Interviewers as Predictors of Participation in HIV Testing

Interviewers have been found to be highly predictive of participation in HIV testing in previous studies, including at HIV surveillance sites such as AHRI (Clark and Houle, 2014; McGovern et al., 2015). Therefore, we also examine whether the interviewers influence the likelihood of their interviewees participating. Amongst female residents in 2010 there were 78 interviewers, and amongst male residents there were 72 interviewers. The median number of interviews conducted per interviewer (the number of residents from whom consent to test for HIV was sought by the interviewer) was 124.5 for women and 174 for men. The median participation rate per interviewer (the number of residents from whom consent to test for HIV was obtained by the interviewer divided by the number of residents from whom consent to test for HIV was sought by the interviewer) was 33% for men and 34% for women. Good interviewers were equally good at raising participation rates for both men and women. For example, the 25th percentile of interviewer consent is 18% for men and 13% for women, while the 75th percentile for interviewer consent is 45% for men and 50% for women. In order to summarize the effect of having a good interviewer on participation in HIV testing, we ran a logistic regression for participation on an indicator variable for having been interviewed by an interviewer who was over the 75th percentile for participation (interviewer consent rates in this regression were calculated as the leave-one-out rate where the individual interviewee was excluded from the numerator and denominator when calculating their interviewer's participation rate in order to avoid a mechanical correlation between the dependent variable and interviewer participation rates), adjusting for the other covariates used in the main analysis. We find an odds ratio for consent of having a good interviewer of 2.1, i.e. having a good interviewer doubled the probability that the interviewee would consent to participate in testing.

Without further data, it is difficult to further separate out the effectiveness of interviewers from the characteristics of their interviewees. In Table A2 we collapse the data at the interviewer level, and regress interviewer effectiveness (defined as the proportion of their interviewees who consented to test) on the characteristics of their interviewees to determine whether better interviewers are systematically associated with particular factors. Because of the simultaneity inherent in this relationship, as well as the fact that we only have 60 observations at the interviewer level (once we collapse those who conducted few interviews), we interpret the following results with caution. There are relatively few interviewees in the categories for marital status and household fuel type, leaving only rural location and some of the distance variables as statistically significant predictors of interviewer success. Collecting or making available additional metadata on interviewer characteristics (such as age, education, and experience) would be helpful for augmenting this analysis.

Table A3 presents a similar analysis for gift voucher receipt (a linear probability model for whether the respondent lived in a household which received a voucher as a function of individual-level characteristics.)

Table A1: 2010 AHRI Surveillance Cohort Descriptive Statistics

	Median	Mean	SD	N
Participated in HIV Testing	0	0.402	0.49	27,684
HIV Positive	0	0.236	0.425	11,117
Male	0	0.388	0.487	27,684
Received Gift Voucher	0	0.07	0.255	27,684
Household Has Piped Water	1	0.595	0.491	27,684
Household Has Flush Toiled	0	0.074	0.262	27,684

**Type of Location**	No.	%	**Household has Electricity**	No.	%
Peri-Urban	8,404	30.36	Yes	17,452	63.04
Rural	17,205	62.15	No	5,156	18.62
Urban	2,075	7.5	N/A	4,483	16.19
Total	27,684	100	Unknown	593	2.14
			Total	27,684	100
**Month of Interview in 2010**					
January	1,319	4.76	**Household Fuel Type**		
February	3,283	11.86	Electricity	13,473	48.67
March	3,996	14.43	Coal or Wood	6,856	24.77
April	2,995	10.82	Gas	1,319	4.76
May	3,334	12.04	Other	918	3.32
June	908	3.28	Unknown	4,478	16.18
July	1,223	4.42	N/A	640	2.31
August	2,949	10.65	Total	27,684	100
September	2,278	8.23			
October	2,460	8.89	**Household Asset Index Quintile**		
November	2,505	9.05	Lowest	4,402	15.9
December	434	1.57	2nd Lowest	4,520	16.33
Total	27,684	100	Middle	4,649	16.79
			2nd Highest	4,624	16.7
**Marital Status**			Highest	4,210	15.21
Married	4,099	14.81	Missing	5,279	19.07
Polygamous	579	2.09	Total	27,684	100
Divorced/Separated/Widowed	2,827	10.21			
Engaged	472	1.7	**Education**		
Never Married	14,987	54.14	None	2,903	10.49
Under Legal Age	4,315	15.59	Primary	2,817	10.18
Unknown/Other	405	1.46	Junior Secondary	4,480	16.18
Total	27,684	100	Upper Secondary	10,255	37.04
			Don't Know	2,112	7.63
**Mother is Alive**			Unknown	5,117	18.48
Dead	22,464	81.14	Total	27,684	100
Alive	4,734	17.1			
Unknown	486	1.76	**Age Group**		
Total	27,684	100	15-19	5,247	18.95
			20-24	4,434	16.02
**Father is Alive**			25-29	3,185	11.5
Dead	21,861	78.97	30-34	2,246	8.11
Alive	5,207	18.81	35-39	2,005	7.24
Unknown	616	2.23	40-44	1,790	6.47
Total	27,684	100	45-49	1,835	6.63
			50-54	1,717	6.2
**Distance to Nearest Clinic**			55-59	1,221	4.41
0-1 Km	3,779	13.65	60+	4,004	14.46
1-2 KM	6,383	23.06	Total	27,684	100
2-3 KM	5,718	20.65			
3-4 KM	4,948	17.87	**Distance to Nearest Secondary School**		
4-5 KM	2,962	10.7	0-1 Km	6,717	24.26
5 KM+	3,894	14.07	1-2 KM	9,730	35.15
Total	27,684	100	2-3 KM	6,913	24.97
			3-4 KM	2,676	9.67
**Distance to Nearest Primary School**			4-5 KM	1,005	3.63
0-1 Km	12,282	44.36	5 KM+	643	2.32
1-2 KM	12,087	43.66	Total	27,684	100
2-3 KM	2,876	10.39			
3 KM+	439	1.59	**Distance to Nearest Level 2 Road**		
Total	27,684	100	0-1 Km	12,204	44.08
			1-2 KM	7,716	27.87
**Distance to Nearest Level 1 Road**			2-3 KM	4,416	15.95
0-1 Km	7,268	26.25	3-4 KM	2,282	8.24
1-2 KM	2,985	10.78	4-5 KM	729	2.63
2-3 KM	1,410	5.09	5 KM+	337	1.22
3-4 KM	1,219	4.4	Total	27,684	100
4-5 KM	1,370	4.95			
5 KM+	13,432	48.52			
Total	27,684	100			

Note to Table A2: An OLS model for the proportion of an interviewer's interviewees who consented to test is shown. The regression is at the interviewer level (one observation per interviewer, with interviewers who conducted fewer than 50 interviewers collapsed into one category). Categorical variables measure the proportion of each interviewer's interviewees in that category, while distance and age variables are measured in average KM and years, respectively.

Table A2: Predictors of Interviewer Success (Interviewer Level)

**Variables**	**Interviewer Consent Rate**	**Variables**	**Interviewer Consent Rate**
**Male**	0.175	**Education (Omitted=None)**	
	(0.142)	Primary	1.097
**Marital Status (Omitted=Married)**			(2.339)
Polygamous	-0.906	Junior Secondary	0.670
	(2.157)		(1.262)
Divorced/Separated/Widowed	0.675	Upper Secondary	-1.227
	(0.909)		(1.385)
Engaged	3.616**	Don't Know	-2.387
	(1.504)		(1.476)
Never Married	0.081	Missing	-3.264
	(0.533)		(1.911)
Under Legal Age	0.410		
	(0.897)	**Flush Toilet Access**	1.442
Missing/Other	-1.904		(1.164)
	(2.969)		
**Household Location (Omitted=Peri-urban)**		**Piped Water Access**	-0.594
Rural	0.734***		(0.486)
	(0.221)		
Urban	0.928	**Distance to Nearest Clinic (KM)**	-0.015
	(1.152)		(0.070)
**Household Electricity (Omitted=Yes)**		**Distance to Nearest Secondary School (KM)**	0.219*
No	-2.309		(0.110)
	(1.411)	**Distance to Nearest Primary School (KM)**	-0.615***
N/A	4.245		(0.153)
	(3.353)	**Distance to Nearest Level 1 Road (KM)**	-0.020
Missing	-9.670*		(0.014)
	(5.481)	**Distance to Nearest Level 2 Road (KM)**	0.314***
**Household Fuel Type (Omitted=Electric)**			(0.101)
Coal or Wood	-0.624		
	(0.477)	**Age**	-0.021
Gas	-1.325		(0.012)
	(1.402)		
Other	-3.512*	**Household Asset Index Quintile (Omitted=1)**	
	(1.806)	2	-0.263
Missing	-2.751		(0.985)
	(4.245)	3	-1.999
Unknown	8.466		(1.562)
	(6.152)	4	-0.600
			(1.615)
**Constant**	1.716	5	-1.840
	(2.492)		(2.082)
		Missing	-0.662
**Observations**	60		(4.246)
**R-squared**	0.923		

Robust standard errors in parentheses
*** p<0.01, ** p<0.05, * p<0.1

Note to Table A3: A linear probability model for whether the individual lived in a household which received the voucher is shown. Standard errors are clustered at the household level. Almost all vouchers were disbursed among households which were contacted in October and November.

Table A3: Predictors of Voucher Receipt (Individual Level)

Variables	Voucher Received	Variables	Voucher Received
**Male**	-0.001	**Education (Omitted=None)**	
	(0.001)	Primary	0.001
**Marital Status (Omitted=Married)**			(0.001)
Polygamous	-0.003	Junior Secondary	0.000
	(0.002)		(0.001)
Divorced/Separated/Widowed	-0.001	Upper Secondary	0.002
	(0.001)		(0.001)
Engaged	-0.004	Don't Know	-0.001
	(0.004)		(0.002)
Never Married	-0.001	Missing	-0.005
	(0.002)		(0.003)
Under Legal Age	-0.000	**Distance to Nearest Clinic (Omitted=0-1KM)**	
	(0.002)	1-2 KM	-0.001
Missing/Other	-0.014		(0.002)
	(0.012)	2-3 KM	0.001
**Month Contacted (Omitted=January)**			(0.001)
February	-0.000	3-4 KM	-0.000
	(0.000)		(0.001)
March	0.001	4-5 KM	0.001
	(0.001)		(0.001)
April	-0.000	5 KM+	0.001
	(0.001)		(0.001)
May	-0.000	**Distance to Nearest Secondary School (Omitted=0-1KM)**	
	(0.000)	1-2 KM	-0.001
June	-0.000		(0.001)
	(0.000)	2-3 KM	0.001
July	-0.000		(0.001)
	(0.001)	3-4 KM	0.001
August	0.000		(0.001)
	(0.000)	4-5 KM	-0.001
September	0.000		(0.001)
	(0.001)	5 KM+	-0.002*
October	1.000***		(0.001)
	(0.001)	**Distance to Nearest Primary School (Omitted=0-1KM)**	
November	0.999***	1-2 KM	0.001
	(0.001)		(0.001)
December	0.500***	2-3 KM	0.000
	(0.027)		(0.001)
**Household Location (Omitted=Peri-urban)**		3 KM +	0.003
Rural	-0.001		(0.003)
	(0.001)	**Distance to Nearest Level 1 Road (Omitted=0-1KM)**	
Urban	0.006	1-2 KM	-0.003
	(0.004)		(0.003)
**Mother Alive(Omitted=No)**		2-3 KM	-0.001
Alive	-0.001		(0.001)
	(0.001)	3-4 KM	0.000
Missing	0.010		(0.002)
	(0.012)	4-5 KM	-0.001
**Father Alive(Omitted=No)**			(0.002)
Alive	0.000	5 KM+	-0.001
	(0.001)		(0.002)
Missing	0.008	**Distance to Nearest Level 2 Road (Omitted=0-1KM)**	
	(0.008)	1-2 KM	-0.000
**Household Electricity (Omitted=Yes)**			(0.001)
No	0.000	2-3 KM	-0.001
	(0.001)		(0.001)
N/A	0.004*	3-4 KM	0.002
	(0.002)		(0.003)
Missing	-0.001	4-5 KM	0.006
	(0.004)		(0.005)
**Household Fuel Type (Omitted=Electric)**		5 KM+	-0.001
Coal or Wood	-0.001		(0.002)
	(0.001)	**Age Group (Omitted=¡20)**	
Gas	-0.000	20-24	0.000
	(0.001)		(0.002)
Other	-0.001	25-29	-0.001
	(0.002)		(0.002)
Missing	0.002	30-34	-0.003
	(0.002)		(0.002)
Unknown	0.000	35-39	-0.003
	(0.002)		(0.002)
**Household Asset Index Quintile (Omitted=1)**		40-44	0.001
2	-0.001		(0.002)
	(0.001)	45-49	0.001
3	0.001		(0.002)
	(0.001)	50-54	0.001
4	-0.000		(0.002)
	(0.002)	55-59	0.002
5	-0.002		(0.002)
	(0.002)	60+	0.002
Missing	-0.002*		(0.002)
	(0.001)		
		**Constant**	0.003
**Flush Toilet Access**	-0.004		(0.003)
	(0.004)		
**Piped Water Access**	0.000	**Observations**	27,684
	(0.001)	**R-squared**	0.973

Clustered standard errors in parentheses
*** p<0.01, ** p<0.05, * p<0.1

Further Details of the Copula Approach

Following Marra et al. (2017), we model the joint distribution of the error terms using copulae. A major advantage of this approach is that these models can be estimated in a standard maximum likelihood framework, resulting in consistent, efficient and asymptotically normal estimators (Smith, 2003), with the log-likelihood (abstracting from the interviewer subscript) given by:

$$\sum_{i=1}^{n} Consent_i \times HIV_i log(p_{11i}) + Consent_i \times (1 - HIV_i) log(p_{01i}) + (1 - Consent_i) log(p_{0i}) \qquad (1)$$

Where p_{0i} is the probability of declining to test, $P(Consent_i = 0)$, p_{11i} is the probability of being HIV positive and consenting to test, $P(Consent_i = 1, HIV_i = 1)$, p_{01i} is the probability of being HIV negative and consenting to test, $P(Consent_i = 1, HIV_i = 0)$. Copula functions can be incorporated into the likelihood function to map multivariate distributions to their marginal distributions. In this case, we are concerned with the copula mapping function C that links the two-dimensional cumulative density function for HIV status and consent to test to the relevant one dimensional margins, $F(HIV, Consent) = C(F_{HIV}(HIV), F_{Consent}(Consent); \theta)$, where θ is an association parameter indicating the degree of dependence.

A number of copulae have been proposed, each with different dependence structures, including, for example, the Frank, Gumbel, Clayton and Joe copulae. While the Frank copula is similar to the Gaussian, the Gumbel, Clayton and Joe are asymmetric, allowing the case where those who are most likely to be HIV negative do not have a greater dependence to test than those who are moderately likely to be HIV negative, while those who are most likely to be HIV positive are those who are the least likely to test. In addition, the rotated versions (90 degrees, 180 degrees, and 270 degrees) of these copula are easily obtained and allow for greater density in either tail of the distribution (Brechmann and Schepsmeier, 2013).

In the standard bivariate probit selection model, the error terms are assumed to be independent and identically distributed (i.i.d.) with means equal to zero, constant variances equal to one, and covariance (correlation coefficient) ρ. Therefore, ρ is the key parameter in the model (u_i and ϵ_i), and if those who are HIV positive are less likely to participate in testing (conditional on observed characteristics), we expect negative dependence. In the copula selection models, a nonparametric measure of association, such as Kendall's Tau (τ) or the gamma association measure (shown in Tables 2 and 3 in the main text), is more appropriate as the dependence modelled by copulae is typically non-linear.

By implementing the selection model allowing for a variety of different forms of dependence, the copula approach allows us to establish whether the results from the standard bivariate probit selection model are sensitive to the assumption of joint normality. Bivariate normality places restrictions on the dependence structure, for example it assumes that the propensity to participate given HIV status (conditional on covariates) is symmetric. Along with the difficulty finding viable exclusions restrictions, this parametric formulation is likely an impediment to the wider use of selection models for dealing with missing data because it cannot be verified and can be seen as arbitrary (Vytlacil, 2002). As these models are estimated under the standard maximum likelihood framework, we are able to use standard diagnostic tools for model fit, and choose our preferred copula specification based on information criteria.

Coupled with plausible exclusion restrictions based on survey design, relaxing the parametric assumptions required for identification in selection models for binary outcomes should remove an impediment to wider use of selection models for dealing with missing data without requiring the undesirable and untestable assumption of missing at random. Moreover, as we discuss in the main text, the copula approach is a viable means of relaxing the bivariate normality assumption when dealing with dichotomous dependent variables and the intercept is a parameter of interest. Even when the outcome of interest is continuous, there are important advantages to the copula method over the semi- and nonparametric approaches. Specifically, the latter require a much larger set of parameters to be estimated, potentially rendering them inefficient and prohibiting a comprehensive set of covariates, and typically involve numerical integration or complex simulation procedures. In contrast, copula models can be estimated under a conventional maximum likelihood framework, allowing, for example, model selection to be based on standard information criteria (Pigini, 2015).

References

S. Baird, E. Chirwa, C. McIntosh, and B. Özler. The short-term impacts of a schooling conditional cash transfer program on the sexual behavior of young women. *Health Economics*, 19(S1):55–68, 2010.

T. Bärnighausen, F. Tanser, A. Malaza, K. Herbst, and M.-L. Newell. HIV status and participation in HIV surveillance in the era of antiretroviral treatment: a study of linked population-based and clinical data in rural south africa. *Tropical Medicine & International Health*, 17(8):e103–e110, 2012.

C. Beyrer, S. Baral, D. Kerrigan, N. El-Bassel, L.-G. Bekker, and D. D. Celentano. Expanding the space: Inclusion of most-at-risk populations in HIV prevention, treatment, and care services. *Journal of Acquired Immune Deficiency Syndromes*, 57(Suppl 2):S96, 1999.

D. E. Bloom and A. S. Mahal. Does the AIDS epidemic threaten economic growth? *Journal of Econometrics*, 77(1):105–124, 1997.

E. C. Brechmann and U. Schepsmeier. Modeling dependence with c-and d-vine copulas: The r-package cdvine. *Journal of Statistical Software*, 52(3):1–27, 2013.

A. Case and C. Paxson. The impact of the AIDS pandemic on health services in Africa: evidence from Demographic and Health Surveys. *Demography*, 48(2):675–697, 2011.

A. Case and C. Paxson. HIV Risk and Adolescent Behaviors in Africa. *American Economic Review*, 103(3): 433–438, 2013.

S. J. Clark and B. Houle. Validation, Replication, and Sensitivity Testing of Heckman-Type Selection Models to Adjust Estimates of HIV Prevalence. *PLOS ONE*, 9(11):e112563, 2014.

D. De Walque. How does the impact of an HIV/AIDS information campaign vary with educational attainment? Evidence from rural Uganda. *Journal of Development Economics*, 84(2):686–714, 2007.

M. S. Fabic, Y. Choi, and S. Bird. A systematic review of Demographic and Health Surveys: data availability and utilization for research. *Bulletin of the World Health Organization*, 90(8):604–612, 2012.

W. Janssens, J. van der Gaag, T. F. Rinke de Wit, and Z. Tanović. Refusal bias in the estimation of HIV prevalence. *Demography*, 51(3):1131–1157, 2014.

K. Kranzer, N. McGrath, J. Saul, A. C. Crampin, A. Jahn, S. Malema, D. Mulawa, P. E. Fine, B. Zaba, and J. R. Glynn. Individual, household and community factors associated with HIV test refusal in rural Malawi. *Tropical Medicine & International Health*, 13(11):1341–1350, 2008.

J. Larmarange, J. Mossong, T. Bärnighausen, and M.-L. Newell. Participation Dynamics in Population-Based Longitudinal HIV Surveillance in Rural South Africa. *PLOS ONE*, 10(4), 2015.

G. Marra, R. Radice, T. Bärnighausen, S. Wood, and M. McGovern. A Simultaneous Equation Approach to Estimating HIV Prevalence with Non-Ignorable Missing Responses. *Journal of the American Statistical Association*, 518(12):484–496, 2017.

M. McGovern, G. Marra, R. Radice, D. Canning, M.-L. Newell, and T. Bärnighausen. Adjusting for Non-Participation Bias at an HIV Surveillance Site in Rural South Africa. *Journal of the International AIDS Society*, 18:19954, 2015.

V. Mishra, B. Barrere, R. Hong, and S. Khan. Evaluation of bias in HIV seroprevalence estimates from national household surveys. *Sexually Transmitted Infections*, 84(Suppl 1):i63–i70, 2008.

F. Obare. Nonresponse in repeat population-based voluntary counseling and testing for HIV in rural Malawi. *Demography*, 47(3):651–665, 2010.

R. Parker and P. Aggleton. HIV and AIDS-related stigma and discrimination: a conceptual framework and implications for action. *Social Science & Medicine*, 57(1):13–24, 2003.

C. Pigini. Bivariate non-normality in the sample selection model. *Journal of Econometric Methods*, 4(1), 2015.

G. Reniers and J. Eaton. Refusal bias in HIV prevalence estimates from nationally representative seroprevalence surveys. *AIDS*, 23(5):621, 2009.

G. Reniers, T. Araya, Y. Berhane, G. Davey, and E. J. Sanders. Implications of the HIV testing protocol for refusal bias in seroprevalence surveys. *BMC Public Health*, 9(1):163, 2009.

M. D. Smith. Modelling sample selection using archimedean copulas. *Econometrics Journal*, 6(1):99–123, 2003.

F. Tanser, V. Hosegood, T. Bärnighausen, K. Herbst, M. Nyirenda, W. Muhwava, C. Newell, J. Viljoen, T. Mutevedzi, and M.-L. Newell. Cohort profile: Africa centre demographic information system (ACDIS) and population-based HIV survey. *International Journal of Epidemiology*, 37(5):956–962, 2008.

F. Tanser, T. Bärnighausen, E. Grapsa, J. Zaidi, and M.-L. Newell. High coverage of ART associated with decline in risk of HIV acquisition in rural KwaZulu-natal, south africa. *Science*, 339(6122):966–971, 2013.

E. Vytlacil. Independence, monotonicity, and latent index models: An equivalence result. *Econometrica*, 70 (1):331–341, 2002.
